# Supplementary material for: End Sequence Analysis Toolkit (ESAT) expands the extractable information from single-cell RNA-seq data
Source: Genome Res. 2016 Oct;26(10):1397–410. doi: 10.1101/gr.207902.116 (PMC5052061; doi:10.1101/gr.207902.116)
Supplement: Supplemental Material [file supp_gr.207902.116_Supp_Materials.tar.gz › ESAT-esat_v0.1/doc/html/classbroad_1_1core_1_1hmm_1_1_markov_model_3_01_t_01_4-members.html]

ESAT: Member List


ESAT


- Main Page
- Related Pages
- Packages
- Classes
- Files

- Class List
- Class Index
- Class Hierarchy
- Class Members

All Classes Namespaces Files Functions Variables Enumerator Pages

- broad
- core
- hmm
- MarkovModel< T >

broad.core.hmm.MarkovModel< T > Member List

This is the complete list of members for broad.core.hmm.MarkovModel< T >, including all inherited members.

|  |  |  |
| --- | --- | --- |
| addState(MarkovState< T > state) | broad.core.hmm.MarkovModel< T > |  |
| addState(MarkovState< T > state, int stateIdx) | broad.core.hmm.MarkovModel< T > |  |
| computePathLogLikelihood(List< T > observationSequence, short[] path, int initialStateIdx, int endStateIdx) | broad.core.hmm.MarkovModel< T > |  |
| drawInitialState() | broad.core.hmm.MarkovModel< T > | protected |
| drawNextState(int prior) | broad.core.hmm.MarkovModel< T > | protected |
| emitPath(int numOfSteps) | broad.core.hmm.MarkovModel< T > |  |
| getConsistencyProblems() | broad.core.hmm.MarkovModel< T > | protected |
| getInitialStateTransitionProbabilities() | broad.core.hmm.MarkovModel< T > | protected |
| getState(String stateName) | broad.core.hmm.MarkovModel< T > |  |
| getStates() | broad.core.hmm.MarkovModel< T > | protected |
| getStateTransitionMatrix() | broad.core.hmm.MarkovModel< T > | protected |
| MarkovModel(int numberOfStates) | broad.core.hmm.MarkovModel< T > |  |
| runBackwardAlgorithm(List< T > observationSequence, ForwardResult< T > forwardData) | broad.core.hmm.MarkovModel< T > |  |
| runForwardAlgorithm(List< T > observationSequence) | broad.core.hmm.MarkovModel< T > |  |
| runForwardAlgorithmNoRescaling(List< T > observationSequence) | broad.core.hmm.MarkovModel< T > |  |
| setEndStateTransitionProbability(int stateIdx, double probability) | broad.core.hmm.MarkovModel< T > |  |
| setInitialStateTransitionProbability(int stateIdx, double probability) | broad.core.hmm.MarkovModel< T > |  |
| setStateTransitionProbability(int stateIdx1, int stateIdx2, double probability) | broad.core.hmm.MarkovModel< T > |  |
| viterbiMostLikelyEstimation(List< T > observationSequence) | broad.core.hmm.MarkovModel< T > |  |


---

Generated on Fri Aug 15 2014 15:10:52 for ESAT by  

 1.8.7
